# Dense granule protein 3 of *Toxoplasma gondii* plays a crucial role in the capability of the tissue cysts of the parasite to persist in the presence of anti-cyst CD8^+^ T cells during the chronic stage of infection

**DOI:** 10.3389/fimmu.2023.1272221

**Published:** 2023-10-05

**Authors:** Rajesh Mani, Mohamed H. Abdelaziz, Eri Ochiai, Qila Sa, Barbara A. Fox, David J. Bzik, Yasuhiro Suzuki

**Affiliations:** ^1^ Department of Microbiology, Immunology and Molecular Genetics, University of Kentucky College of Medicine, Lexington, KY, United States; ^2^ Deaprtment of Microbiology and Immunology, Geisel School of Medicine at Dartmouth, Lebanon, NH, United States

**Keywords:** *Toxoplasma gondii*, cyst, dense granule protein, rhoptry protein, immune evasion

## Abstract

*Toxoplasma gondii* establishes chronic infection by forming tissue cysts, and this chronic infection is one of the most common parasitic infections in humans. Our recent studies revealed that whereas CD8^+^ T cells of genetically resistant BALB/c mice have the capability to remove the tissue cysts of the parasite through their perforin-mediated activities, small portions of the cysts are capable of persisting in the presence of the anti-cyst CD8^+^ T cells. It is currently unknown how those small portions of the cysts resist or escape the T-cell immunity and persist in the hosts. In the present study, we discovered that the cysts, which persisted in the presence of the perforin-mediated CD8^+^ T-cell immunity, have significantly greater mRNA levels for four dense granule proteins, GRA1, GRA2, GRA3, and GRA7, and one rhoptry protein, ROP35, than the total population of the cysts present in the absence of the T cells. In addition, increased levels of mRNA for GRA1, GRA3, and ROP35 in the cysts significantly correlated with their successful persistence through the condition in which greater degrees of reduction of the cyst burden occurred through anti-cyst CD8^+^ T cells. In addition, GRA3-deficient *T. gondii* displayed significantly enhanced elimination of the cysts by anti-cyst CD8^+^ T cells when compared to the wild-type parasite. These results indicate that GRA3 is a key molecule that mediates in the capability of *T. gondii* cysts to persist by resisting or evading the anti-cyst activity of CD8^+^ T cells during the later stage of infection.

## Introduction

*Toxoplasma gondii* is an obligate intracellular protozoan parasite capable of establishing a persisting chronic infection. This chronic infection is widespread in humans worldwide, with one-third of the population being estimated to be infected (1). During the acute stage of the infection, IFN-γ-mediated protective immunity controls the proliferation of tachyzoites (the acute stage form of the parasite) (2). However, a part of the tachyzoites differentiates into tissue cysts in various organs, especially in the brain, and establishes chronic infection. Since individuals chronically infected with *T. gondii* usually remain seropositive for this parasite for decades or possibly life, it was generally considered that the immune system is unable to recognize or target the cyst stage of this parasite. However, our recent studies uncovered that CD8^+^ T cells have the capability to remove *T. gondii* cysts through a perforin-mediated mechanism from the brains of chronically infected BALB/c mice (3–5), which are genetically resistant to the infection (6, 7). However, even in the presence of the anti-cyst T-cell immunity, small numbers of cysts still persist in the infected hosts. It is currently unknown how these *T. gondii* cysts are able to avoid their elimination by the CD8^+^ T cell-mediated protective immunity. Since there is currently no drug available to target the cyst stage of *T. gondii*, it is crucial to elucidate the mechanisms by which *T. gondii* cysts persist in the presence of the anti-cyst protective immunity and generate the basis for developing a method that disrupts the persisting mechanism(s) of the cysts for their eradication.

The rhoptry and the dense granules are two major secretory organelles of *T. gondii*, which are critical for the pathogenesis of this parasite. The rhoptry proteins (ROPs) are secreted to assist the invasion of tachyzoites into host cells (8). Dense granule proteins (GRAs) are secreted after their invasion into host cells and support the formation of the parasitophorous vacuoles (PV), in which the parasite resides and proliferates within infected cells. When the intracellular tachyzoites transform to bradyzoites for developing tissue cysts, the PV transforms to the cyst wall (9). Since the bradyzoites maintain expressions of GRA and ROP proteins (9, 10), and since some of GRA and ROP proteins have been shown to disrupt the effector mechanisms of IFN-γ-mediated protective immunity against tachyzoites (11–13), it would be possible that certain GRA and/or ROP proteins mediate the evasion of *T. gondii* cysts from the perforin-mediated anti-cyst CD8^+^ T-cell immunity.

In the present study, we examined mRNA expression levels for eight GRAs (GRA1–GRA8) and five ROPs (ROP5, ROP16, ROP17, ROP18, and ROP35) in *T. gondii* cysts that persisted in the presence of anti-cyst CD8^+^ T cells. We found that mRNA levels for GRA1, GRA2, GRA3, GRA7, and ROP35 are significantly greater in *T. gondii* cysts that persisted in the presence of the perforin-mediated anti-cyst CD8^+^ T cells than a total population of cysts that persisted in the absence of those T cells. We further identified that tissue cysts of the GRA3-deficient mutant strain of *T. gondii* have a significantly increased susceptibility to anti-cyst activity of CD8^+^ T cells and display enhanced removal by the T cells. Thus, the present study revealed that GRA3 plays a crucial role in the capability of *T. gondii* cysts to persist by resisting or evading the anti-cyst activity of CD8^+^ T cells in chronically infected hosts.

## Materials and methods

### Mice

BALB/c-background SCID, which lack T cells, and RAG1-knockout (RAG1^−/−^) mice, which lack both T and B cells, and wild-type (WT) BALB/c mice were from the Jackson Laboratory (Bar Harbor, ME). BALB/c-background perforin-deficient (Prf1^−/−^) mice (14) were originally provided by John T. Harty (University of Iowa) and bred in our animal facility. Female mice were used for all studies. SCID or RAG1^−/−^ mice were used as recipients of CD8^+^ T cells from WT or Prf1^−/−^ BALB/c mice. There were three to five mice in each experimental group. Mouse care and experimental procedures were performed under specific pathogen-free conditions in accordance with established institutional guidance and approved protocols from the Institutional Animal Care and Use Committee.

### *T. gondii* strains

The ME49 strain of *T. gondii* was maintained by infecting Swiss-Webster mice intraperitoneally with 10 cysts obtained from the brains of chronically infected mice of this strain (3–5). The WT and GRA3-deficient (ΔGRA3) Prugniaud (Pru) strains of the parasite were maintained as tachyzoites in their cultures with monolayers of human foreskin fibroblasts in DMEM medium (Gibco/Thermo Fisher Scientific, Waltham, MA) containing 10% fetal bovine serum (Gibco). Both the ME49 and Pru strains belong to the genotype II of *T. gondii*.

### Infection of mice with *T. gondii*

Mice were infected with 10 cysts (for T-cell donors) or 20 cysts (for T-cell recipients) of the ME 49 strain of *T. gondii* orally by gavage. SCID and RAG1^−/−^ mice were treated with sulfadiazine (Sigma-Aldrich, St. Louis, MO) in the drinking water (400 mg/L) beginning at 9 days after infection for the entire periods of the experiments to control proliferation of tachyzoites and establish a chronic infection (5, 15). The Prf1^−/−^ mice received sulfadiazine in the same manner beginning at 26 days after infection. In a part of the experiments, SCID mice were infected intraperitoneally with 2 × 10^3^ tachyzoite of the WT or ΔGRA3 Pru strains (16) and treated with sulfadiazine in the drinking water beginning at 7 days after infection to establish chronic infection, and sulfadiazine dose was increased to 1 g/L in drinking water from 13 days after infection to maintain the chronic stage of the infection.

### Purification and transfer of CD8^+^ T cells

Spleen cells were obtained from WT and Prf1^−/−^ mice chronically infected with the ME49 strain and suspended in Hanks’ balanced salt solution (Hyclone, Logan, UT) with 2% fetal bovine serum (Sigma). CD8^+^ immune T cells were purified from the spleen cells using MACS with microbeads-conjugated anti-mouse CD8 (53-6.7) monoclonal antibody (Miltenyi Biotec, Auburn, CA) (5, 17). As a control, CD8^+^ normal T cells were purified from the spleens of uninfected WT mice in the same manner. Infected SCID and RAG1^−/−^ mice received the purified CD8^+^ T cells (2.1–3.5 × 10^6^ cells) intravenously from a tail vein at 3 weeks after infection (5).

### Quantification of cyst numbers in the brain

At 1 day before (Day −1) or 7 days after (Day 7) the transfer of CD8^+^ normal or immune T cells, half of each brain of infected SCID and RAG1^−/−^ mice was triturated with a mortar and pestle in 0.5 mL of PBS (3, 5), and the number of cysts in at least three aliquots (20 μl each) of each brain suspension was counted microscopically.

### Quantification of amount of mRNA for selected *T. gondii* molecules

RNA was purified from half of each brain of infected SCID and RAG1^−/−^ mice obtained at Day −1 or Day 7 of the CD8^+^ T-cell transfer, and the amount of mRNA for 13 secretory molecules (GRA1–8, ROP5, ROP16–18, and ROP35), bradyzoite antigen 1 (BAG1), and bradyzoite-specific surface antigen 2C (SAG2C) was measured by reverse transcription real-time PCR (RT-PCR) using the StepOne Plus real-time PCR system with TaqMan reagents (Allied Biosystems, Norwalk, CT) (5, 15). BAG1 was used as a molecule constitutively expressed only in the bradyzoites stage of *T. gondii*, and its mRNA levels were used to indicate the cyst burden in the brains of infected mice. SAG2C is a bradyzoite-specific molecule expressed on the surface of the bradyzoites and used as a control molecule for comparison with the secretory GRA and ROP molecules. In infection with the WT Pru and ΔGRA3 strains of *T. gondii*, mRNA levels for GRA3 were also measured to confirm the absence of GRA3 mRNA in the brains of mice infected with the ΔGRA3 strain. Sequences of the primers and probe for each of these *T. gondii* molecules are described in **Table 1**. Relative expression levels of GRA1–8, ROP5, ROP16–18, ROP35, and SAG2C in the cysts were calculated as ratios of their mRNA levels to BAG1 mRNA levels. The mRNA levels for mouse CD8β and Prf1 were also measured using ready-made primer and probes from Applied Biosystems.

**Table 1:** Sequences of the primers and probes employed in quantitative real-time RT-PCR for the *T. gondii* molecules examined.

Gene	Forward Primer 5'→ 3'	Reverse Primer 5'→ 3'	Probe 5'→ 3'BAG1
BAG1	TCACGTGGAGACCCAGAGT	CTGGCAAGTCAGCCAAAATAATCAT	TTTGCTGTCGAACTCC
SAG2c	CGCACAGTCATTCAACCAAAAAGTT	TGGAGGTGACCGCTACAGT	TTGTGTCGTTCAGATAAATG
GRA1	ACAGGCAACCCGGACTTG	GACTTCGCTGTACGATCCATCT	ATCGCCATTAAAACTTC
GRA2	GCCAAAGAAGCAGCTGGAA	CTCCACATTCGCGAGTTTCTTG	ACGGTCACCATGCCCC
GRA3	GAGTCGGATAAGGTGGACAATCAG	CAACTCCTCTTCGACCTTCTTCAT	ACGCTCACCTCCCTCC
GRA4	CAGCCTCTTCGCACACAAG	GGGAGGAGGAGCTGCTG	ACGGCCACCTATTATC
GRA5	AAAGTGAAGACCGATCGTTATTCGA	CTGCAGTCCTCACTGGATGTC	CCGCTGCTCTTCCCCT
GRA6	CCGCGCTGGCGAATG	GCCCTGTTCTTCGATTCTTTCCT	CCTCCGACTTCCCC
GRA7	CACGAGACGAAAGGGTGGTT	GCCGCTGTTCTCGACAAAGA	CCTGGCAGCATCACGT
GRA8	CATGCCACAGCCAGAGGTT	CTGGAGTACCCACTGGATATGGA	CCGCCACTTCAGCATC
ROP5	ACTATGGGTGCCGAGAATTCC	GCCGCAAAAGTCGCTTCAT	CAACCGCTCCAGCTCT
ROP16	GAAGAGGGTCTGGAAGAAGTTCAG	GTCCGGAACCGCTACGA	CTGCCGCACAGCTT
ROP17	GCGGCTTTGGTCTTGTGTAC	CCTCATTGTTCATCACCCGTTGA	CCACAGGGCAACCAT
ROP18	TGGCTGTTAAGGTTTTCATGTCAGA	CCTCTGCAAGTCACGCATAGTC	ATCGGTGGGCTCCTTT
ROP35	CACACCTCGTCCAAGTTTCCA	CTTCGCCCCTCTTGTTTCTTG	CCGGACATGTTTCCTG

### Statistical analysis

Levels of difference between experimental groups were determined by one-way ANOVA with Tukey’s multiple comparison test or Mann–Whitney test (GraphPad Prism software, version 9.3.1). Levels of significance in correlations between the degree of increased expression levels of selected GRA and ROP molecules and the successful persistence of *T. gondii* cysts through the condition in which greater portions of cysts from their total populations are eliminated were determined by Pearson test (GraphPad Prism software). The correlations that provided *p* < 0.05 were considered significant.

## Results

### *T. gondii* cysts that persisted in the presence of CD8^+^ immune T cells have increased mRNA levels for five selected GRA and ROP proteins

SCID mice lacking T cells were infected with *T. gondii* and treated with sulfadiazine to establish and maintain the tissue cysts of the parasite in their brains (3–5). At 3 weeks after infection, one mouse group received a systemic transfer of CD8^+^ immune T cells (3.5 × 10^6^ cells) purified from the spleens of WT mice chronically infected with the parasite. Another group of mice received CD8^+^ normal T cells from uninfected WT mice as a control in the same manner. Two additional groups of infected SCID mice did not receive any T cells as another control, and cyst numbers and mRNA levels for bradyzoite (cyst)-specific BAG1 in their brains were measured at 1 day before (Day −1) and 7 days after (Day 7) the T-cell transfer. The cyst burden and BAG1 mRNA levels in the brains of the recipients of the CD8^+^ T cells were measured at Day 7. We also performed an independent experiment using RAG1^−/−^ mice that lack both T and B cells as the recipients of the CD8^+^ T cells (3.2 × 10^6^ cells) from infected and uninfected control WT mice in the same manner.

**Figure 1:**
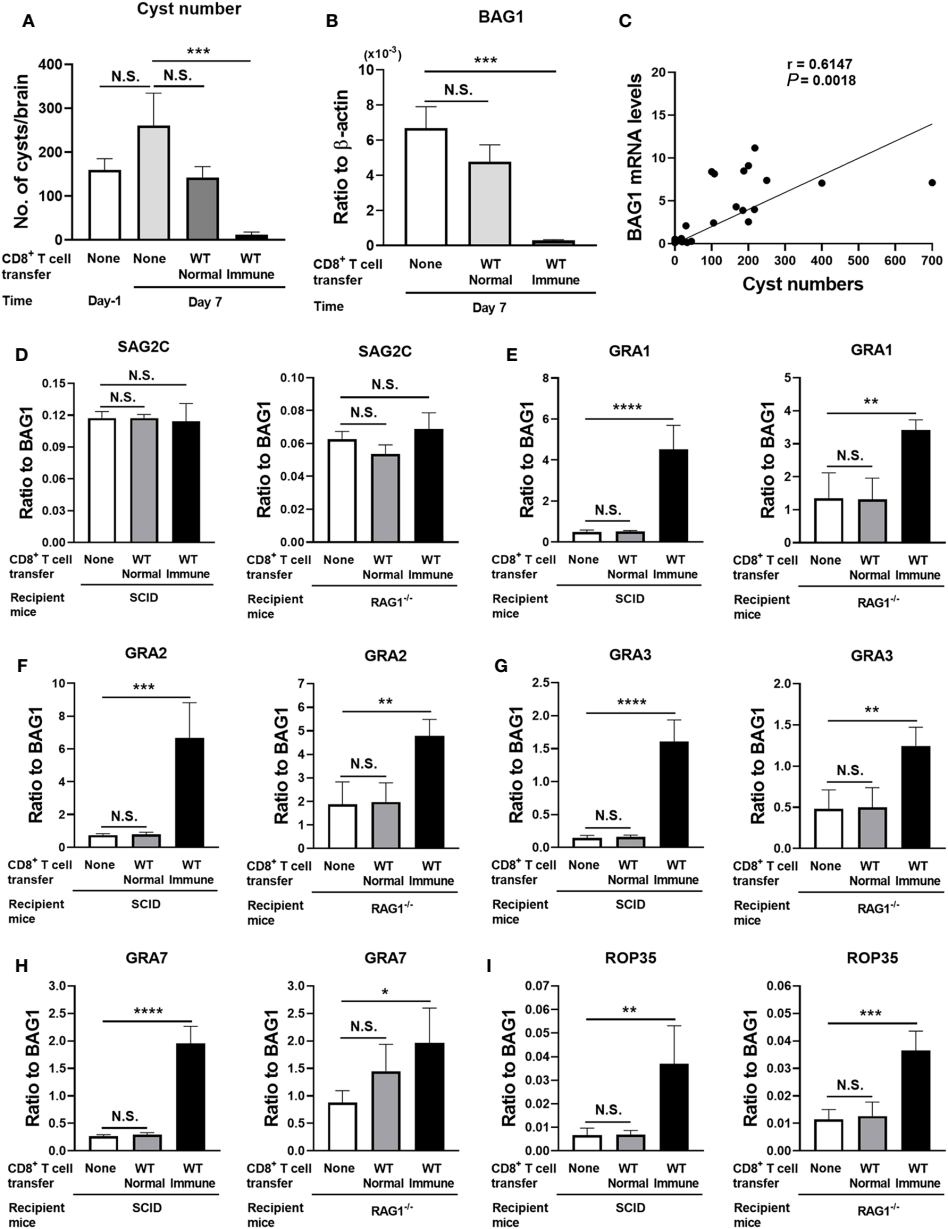
*T. gondii* cysts that persisted in the presence of CD8^+^ immune T cells express increased levels of mRNA for five selected GRA and ROP molecules (GRA1, 2, 3, and 7 and ROP35) when compared to the cysts that existed in the absence of the T cells. SCID mice were infected orally with 20 cysts of the ME49 strain and treated with sulfadiazine in drinking water (400 mg/L) beginning at 9 days after infection for the entire period of the experiment. At 3 weeks after the infection, CD8^+^ immune T cells (3.5 × 10^6^ cells) purified from the spleens of infected WT mice or CD8^+^ normal T cells from uninfected WT mice were injected intravenously from a tail vein into the SCID mice. Two additional groups of infected, sulfadiazine-treated SCID mice did not receive any T cells as a control, and their brains were collected at 1 day before (Day −1) and 7 days after the time of the T-cell transfer. The brains of the infected SCID mice that received the CD8^+^ normal or immune T cells were collected at Day 7. Half of each of these brains was applied for cyst count, and another half was applied for RT-PCR to measure mRNA levels for bradyzoite (cyst)-specific BAG1 and SAG2C, and 13 secretory proteins (8 GRAs [GRA1–8] and 5 ROPs [ROP5, ROP16–18, and ROP35]). We performed an independent replicate study using RAG1^−/−^ mice as the recipients of the CD8^+^ normal and immune T cells in the same manner except for the number of CD8^+^ T cells (3.2 × 10^6^ cells) transferred in this experiment. **(A)** Cyst numbers in the brain, **(B)** cerebral mRNA levels for BAG1, and **(C)** a correlation between the cyst numbers and BAG1 mRNA levels at Day −1 and Day 7 in the control mice and the recipients of the CD8^+^ T cells (data from both SCID and RAG1^−/−^ mice are combined). Relative expression levels of mRNA for **(D)** bradyzoite-specific surface molecule (SAG2C) and **(E–I)** GRA1, GRA2, GRA3, GRA7, and ROP35 in ratios to BAG1 mRNA levels at Day 7 in the brains of SCID and RAG1^−/−^ mice with and without a transfer of CD8^+^ normal or immune T cells. There were four or five mice in each experimental group in each of the studies with SCID and RAG1^−/−^ mice. Data represent the mean ± SEM. *p < 0.05, **p < 0.01, ***p < 0.001, and ****p < 0.0001.* N*.S.: Not significant.

Cyst numbers in the control group without any T-cell transfer did not differ between Day −1 and Day 7, indicating that their cyst numbers were stable during this time period in the absence of T cells (**Figure 1A**, the data from both SCID and RAG1^−/−^ mice are combined). In contrast, the number of cysts in the recipients of the CD8^+^ immune T cells was 21 times less than that in the control mice with no T-cell transfer at Day 7 (*p* < 0.001) (**Figure 1A**). Consistently, cerebral mRNA levels for BAG1 in the CD8^+^ immune T-cell recipients were 27 times less than the control mice with no T-cell transfer at Day 7 (*p* < 0.001) (**Figure 1B**, the data from both SCID and RAG1^−/−^ mice are combined). In contrast, both cyst numbers and BAG1 mRNA levels in the brains of the mice that had received the normal T cells did not differ from those of the control mice without any T-cell transfer (**Figures 1A, B**). Thus, CD8^+^ immune T cells eliminated a majority (95%) of *T. gondii* cysts from the brains of the recipients within 7 days after the transfer of T cells. In other words, a small population (5%) of cysts had successfully persisted despite the presence of the anti-cyst T-cell immunity that had eliminated a majority (95%) of the cysts.

We also examined whether cerebral BAG1 mRNA levels directly correlate with *T. gondii* cysts numbers in the brains of these mice. In this analysis, we applied the BAG1 mRNA level and cyst number of each mouse in all of the experimental groups at Day 7 described above. The BAG1 mRNA levels strongly correlated with the cyst numbers (*p* = 0.0018, **Figure 1C**), indicating that cerebral BAG1 mRNA levels are an effective indicator for cyst burdens in the brains of these mice with and without the transfer of CD8^+^ normal and immune T cells.

We next address a possibility that the small portion (5%) of the cysts, which successfully persisted in the presence of CD8^+^ immune T cells, have a unique molecular expression profile when compared to the total cyst population that persisted in the absence of those T cells in the control group. *T. gondii* has two major groups of secretory proteins, ROPs and GRAs. The ROPs are secreted during an invasion of the parasite into host cells (8), whereas GRAs are secreted after the parasite invaded into host cells to assist the formation of the PV and the cyst wall (9). We examined whether relative mRNA expression levels for eight GRAs (GRA1–8) and five ROPs (ROP5, ROP16–18, and ROP35) differ between the total population of cysts, which existed in the absence of T cells in the control mice, and the small portion of the cysts that successfully persisted for 7 days in the presence of the CD8^+^ immune T cells. Among GRA proteins, GRA1–8 were chosen since they are not only secretory proteins but also known to be present in the cysts and/or the cyst wall detected in the brains of infected mice (18) and their absence often disrupts the cyst wall integrity and/or number of cysts in infected mice (16, 19, 20). Regarding ROP proteins, ROP5, ROP17, ROP18, and ROP35 were chosen, since the absence of any of these molecules results in marked decreases in the number of cysts in infected mice (21). ROP16 was used as a control for the other four ROPs, since a deletion of this molecule rather increases the number of cysts in the brains of infected mice (21). SAG2C was used as a control molecule that is not a secretory protein and expressed on the surface of the bradyzoites present within the cysts (22).

We calculated the relative expression levels of the mRNA levels for each of the eight GRAs, five ROPs, and the control molecule, SAG2C, in the ratios to BAG1 mRNA levels. Since BAG1 mRNA levels indicate cerebral cyst burden in infected mice as shown in **Figures 1A–C**, the relative expression levels of mRNA for the GRAs and ROPs in ratios to BAG1 mRNA levels indicate relative expression levels of these secretory molecules in the cysts present in the brains of these mice.

The relative expression levels of the control molecule, SAG2C, to BAG1 mRNA levels did not differ between the total population of the cysts present in the absence of T cells and the small portion of the cysts that successfully persisted in the presence of the CD8^+^ immune T cells in both SCID and RAG1^−/−^ mice (**Figure 1D**). In contrast, relative expression levels of mRNA for four GRAs (GRA1, GRA2, GRA3, and GRA7) and one ROP (ROP35) consistently showed significantly greater mRNA levels in the cysts that successfully persisted in the presence of the CD8^+^ immune T cells than the cysts that persisted in the absence of any T cells in both SCID and RAG1^−/−^ mice (*p* < 0.05, *p* < 0.01, *p* < 0.001, or *p* < 0.0001, **Figures 1E–I**). In contrast, the relative mRNA expression levels for these five secretory molecules in the cysts that persisted in the recipients of CD8^+^ normal T cells from uninfected mice did not differ from the cysts present in the control mice that did not receive T cells in both SCID and RAG1^−/−^ mice (**Figures 1E–I**). The transfer of the normal T cells did not induce significant reduction of cyst numbers and BAG1 mRNA levels as described earlier (**Figures 1A, B**). These results indicate that the elimination of a majority of *T. gondii* cysts by anti-cyst activity of CD8^+^ immune T cells resulted in a selection of the cysts that express significantly increased mRNA levels for at least the five selected secretory molecules, GRA1, GRA2, GRA3, GRA7, and ROP35, suggesting that these GRA and ROP proteins could mediate in the capability of *T. gondii* cysts to persist in the presence of anti-cyst CD8^+^ T-cell immunity.

### Perforin-mediated activity of CD8^+^ immune T cells is required for the selection of a particular population of *T. gondii* cysts that have increased mRNA expression levels for the five selected GRA and ROP proteins

We previously demonstrated that perforin is required for the activity of CD8^+^ immune T cells to remove *T. gondii* cysts from the brains of infected mice (5, 23). Therefore, we examined whether the persistence of the cysts with upregulated expressions of mRNA for GRA1, GRA2, GRA3, GRA7, and ROP35 occur only when the immune T cells express perforin. Infected and sulfadiazine-treated SCID mice received CD8^+^ immune T cells from chronically infected WT and Prf1^−/−^ mice. As a control, an additional group of infected SCID mice did not receive any T cells.

**Figure 2:**
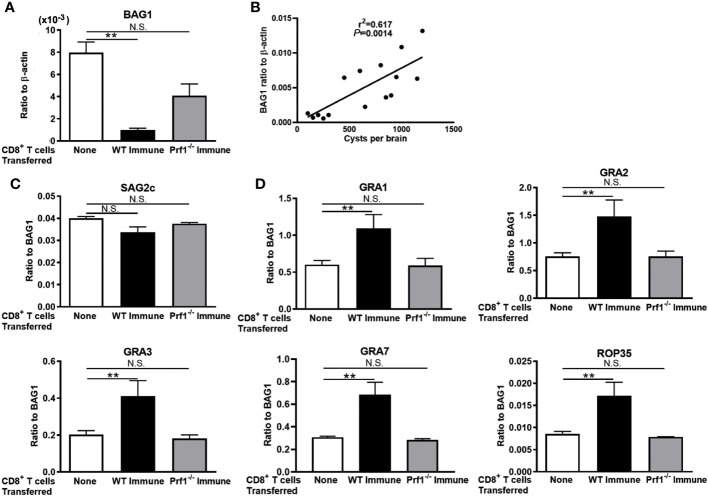
The perforin-mediated activity of CD8^+^ T cells is required for a selection of *T. gondii* cysts that have increased mRNA levels for GRA1, GRA2, GRA3, GRA7, and ROP35 for their persistence in the presence of the T cells. SCID mice were infected orally with 20 cysts of the ME49 strain and treated with sulfadiazine in drinking water (400 mg/L) beginning at 9 days after infection for the entire period of the experiment. At 3 weeks after the infection, CD8^+^ immune T cells (2.1 × 10^6^ cells) purified from the spleens of infected WT or Prf1^−/−^ mice were injected intravenously from a tail vein into the SCID mice. Seven days later, their brains were applied for determining **(A)** mRNA levels for BAG1 and **(B)** a correlation between the cyst numbers and BAG1 mRNA levels at Day 7. The relative expression levels of mRNA for **(C)** SAG2C and **(D)** GRA1, GRA2, GRA3, GRA7, and ROP35 in ratios to BAG1 mRNA levels were also measured. There were three or four mice in each experimental group. Data represent the mean ± SEM. **p < 0.01.* N*.S.: Not significant.

Seven days after the T-cell transfer, BAG1 mRNA levels in the brains of the recipients of WT CD8^+^ T cells were significantly less than those of the control mice that did not receive T cells (*p* < 0.01, **Figure 2A**), whereas BAG1 mRNA levels in the brains of the mice that received Prf1^−/−^ CD8^+^ T cells did not differ from those of the control mice with no T-cell transfer (**Figure 2A**). Their cerebral BAG1 mRNA levels significantly correlated with cyst numbers in their brains (**Figure 2B**), which is consistent with the results shown in **Figure 1C**, confirming that cerebral BAG1 mRNA levels are an effective indicator of the cyst burden in the brain of these mice as well.

We then examined mRNA expression levels for the four selected GRAs (GRA1, GRA2, GRA3, and GRA7), one ROP (ROP35), and one control molecule (SAG2C) of *T. gondii* molecules between the cysts that persisted in the presence and the absence of the perforin-mediated anti-cyst activity in CD8^+^ immune T cells. The relative expression levels of mRNA for the control molecule, SAG2C, in ratios to BAG1 mRNA levels did not differ between the recipients of WT CD8^+^ immune T cells and the control mice that did not receive T cells (**Figure 2C**). Similarly, the ratios of SAG2C mRNA levels to BAG1 mRNA levels did not differ between the recipients of the Prf1^−/−^ T cells and the control mice with no T-cell transfer (**Figure 2C**). In contrast, the relative expression levels of mRNA levels for GRA1, GRA2, GRA3, GRA7, and ROP35 in ratios to BAG1 mRNA levels were markedly and significantly greater in the brains of the recipients of WT CD8^+^ immune T cells than the control mice with no T-cell transfer (*p* < 0.01 for each of these GRA and ROP proteins, **Figure 2D**). Notably, the relative expression levels of mRNA levels for these selected GRA and ROP proteins in ratios to BAG1 mRNA levels did not differ between the recipients of Prf1^−/−^ CD8^+^ T cells and the control mice with no T-cell transfer (**Figure 2D**). These results together indicate that the selection of the cysts with increased mRNA expression for GRA1, GRA2, GRA3, GRA7, and ROP35 occurred when the perforin-mediated anti-cyst activity in CD8^+^ immune T cells is present. Therefore, it would be possible that increased expression of these selected secretory molecules plays important roles in immune evasion of *T. gondii* cysts from the perforin-mediated protective activity of CD8^+^ T cells for the successful survival of those selected population of *T. gondii* cysts.

### Greater expression levels of mRNA for GRA1, GRA3, and ROP35 in *T. gondii* cysts correlate with their successful persistence through the condition in which greater portions of cysts are eliminated from their total population by CD8^+^ T cells

To further address the possibility that increased expressions of GRA1, GRA2, GRA3, GRA7, and ROP35 are involved in the immune evasion of *T. gondii* cysts to successfully persist in the presence of the perforin-mediated anti-cyst CD8^+^ T-cell immunity, we examined whether the degree of increase in the relative mRNA expression levels for GRA1, GRA2, GRA3, GRA7, and ROP35 in the cysts directly correlates with their capability to successfully persist through the immune environment in which greater portions of cysts are eliminated. We applied the data from SCID and RAG1^−/−^ mice that received CD8^+^ T cells from uninfected and infected WT mice and infected Prf1^−/−^ mice shown in **Figures 1** and **2** in this analysis. SAG2C was included as a non-secretory control molecule whose expression level does not correlate with the capability of *T. gondii* cysts to persist in the presence of anti-cyst CD8^+^ immune T cells.

In this analysis, the mean value of the relative mRNA levels of GRA1, GRA2, GRA3, GRA7, and ROP35 in ratios to BAG1 mRNA levels in the control mice that did not receive any T cells was used as the base value, and the fold increases in the relative expression levels of these selected secretory molecules in the cysts that had persisted in the presence of CD8^+^ normal T cells and CD8^+^ immune T cells from infected WT and Prf1^−/−^ mice were calculated as their ratios to their base values in the control mice with no T-cell transfer. The degree of the decrease in the cyst burden was calculated as the fold decreases in cerebral BAG1 levels in the recipients of the normal T cells and immune T cells from infected WT and Prf1^−/−^ mice in comparison with the mean value of BAG1 mRNA levels in the control mice with no T-cell transfer.

**Figure 3:**
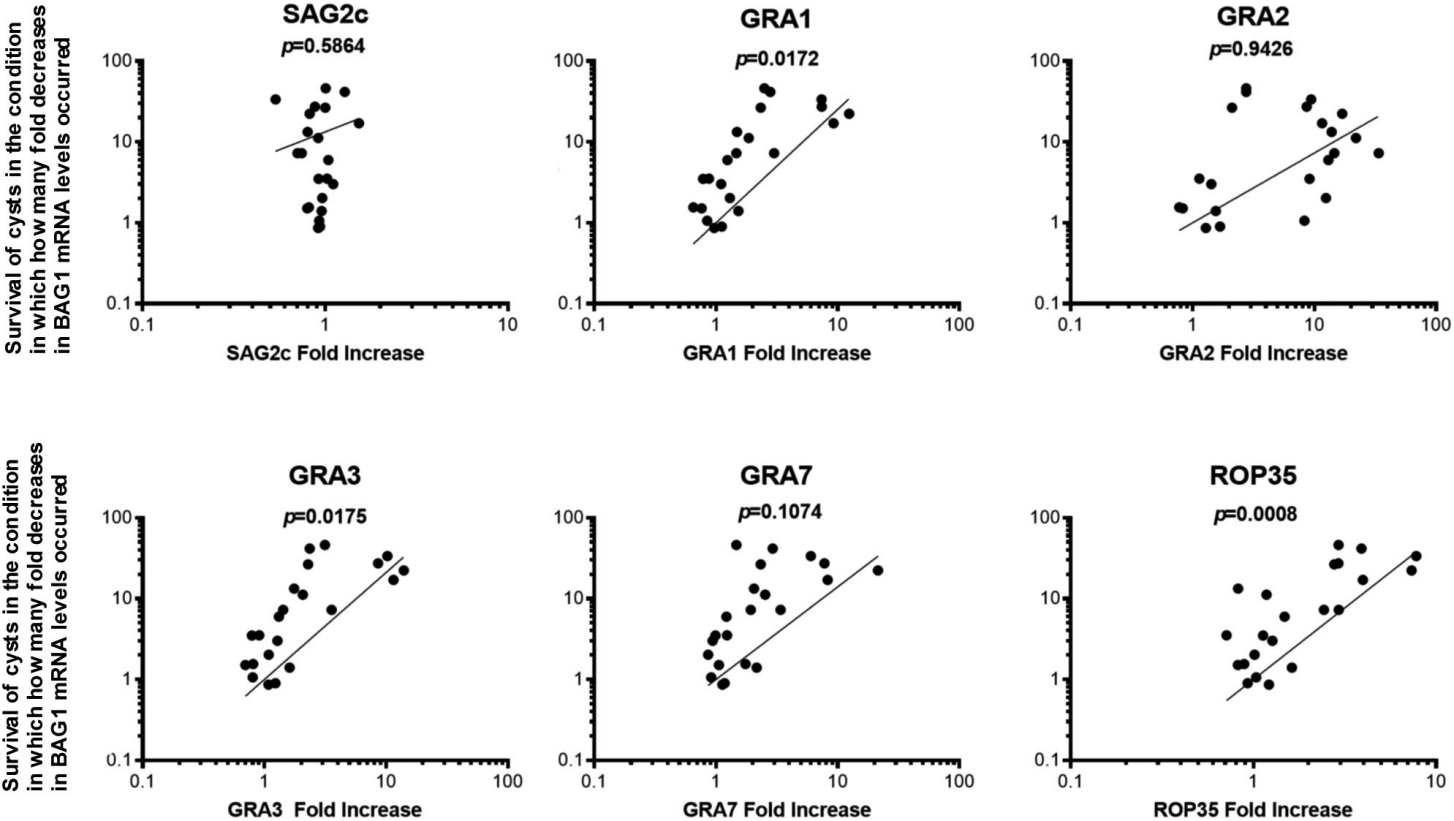
Greater expression levels of GRA1, GRA3, and ROP35 mRNA correlate with the successful persistence of *T. gondii* cysts through the condition in which greater portions of the cysts are eliminated from a total cyst populations by CD8^+^ immune T cells. SCID and RAG1^−/−^ were infected orally with 20 cysts of the ME49 strain and treated with sulfadiazine in drinking water (400 mg/L) beginning at 9 days after infection for the entire period of the experiment. At 3 weeks after the infection, CD8^+^ normal and immune T cells (3.5 and 3.2 × 10^6^ cells) purified from the spleens of normal and infected WT mice were injected intravenously from a tail vein into the SCID and RAG1^−/−^ mice, respectively. In another experiment, SCID mice were infected and treated with sulfadiazine in the same manner and received a systemic transfer of CD8^+^ immune T cells (2.1 × 10^6^ cells) purified from the spleens of infected WT or Prf1^−/−^ mice. The degree of increase in the mRNA expression levels for SAG2C GRA1, GRA2, GRA3, GRA7, and ROP35 in the cysts that persisted in the presence of these T cells was determined by the number of fold increases in their relative expression levels to BAG1 mRNA levels in those cysts in the recipients of CD8^+^ T cells when compared to the cysts that persisted in the control mice that did not receive any T cells. The degrees of elimination of *T. gondii* cysts were determined by the number of fold decreases in the cyst burden (BAG1 mRNA levels) occurred in these T-cell recipients in comparison with the mean value of BAG1 mRNA levels in the control mice that did not receive any T cells.

Greater expression levels of only GRA1, GRA3, and ROP35 in ratios to BAG1 mRNA levels significantly correlated with their persistence through the condition in which greater degrees of reduction of BAG1 mRNA levels occurred in the recipients of the CD8^+^ T cells (*p* < 0.05 for GRA1 and GRA3, and *p* < 0.001 for ROP35, **Figure 3**). GRA7 also shows a tendency of the correlation (**Figure 3**), but the degree of the correlation did not reach significance (*p* = 0.1074, **Figure 3**). GRA2 did not show any trend of the correlation (*p* = 0.9426, **Figure 3**). The degree of increased expression of SAG2C mRNA levels did not correlate with the efficiency of cysts to persist in the presence of the T cells as expected (*p* = 0.5864, **Figure 3**). Thus, GRA1, GRA3, and ROP35 appear to be the most likely candidates for the *T. gondii* molecules that play important roles in the immune evasion of the cyst stage of the parasite to persist in the presence of anti-cyst CD8^+^ immunity in infected hosts.

### Deficiency of GRA3 facilitates an elimination of *T. gondii* cysts by CD8^+^ immune T cells

Among the three molecules, GRA1, GRA3, and ROP35, that showed significant correlations between the degrees of their increased expressions and increased survival rates of *T. gondii* cysts in the presence of CD8^+^ T cells in **Figure 3**, GRA3 has been shown to be present in both cyst wall and the space within the cysts detected in the brains of infected mice (18, 24), and was suggested to play a structural or organizational role during cyst development or in cyst maintenance (16). Thus, we examined whether a deficiency of GRA3 ablates the capability of *T. gondii* cysts to persist in the presence of anti-cyst activity of CD8^+^ immune T cells. Two groups of SCID mice were infected with 2 × 10^3^ tachyzoites of the ΔGRA3 mutant strain, and two additional groups of mice were infected with the WT Pru strain in the same manner. The ΔGRA3 strain was generated from the WT Pru strain (16), which belongs to the genotype II of *T. gondii* as does the ME49 strain that was used in the studies shown in **Figures 1** **–** **3**. The mice received sulfadiazine treatment beginning at 7 days after infection for the entire period of the study. At 3 weeks after infection, one of the two groups of each of the WT Pru- and ΔGRA3-infected SCID mice received CD8^+^ immune T cells (3 × 10^6^ cells) from WT mice infected with the ME49 strain, and another group of each of the WT Pru- and ΔGRA3-infected SCID mice did not receive any T cells as a control.

**Figure 4:**
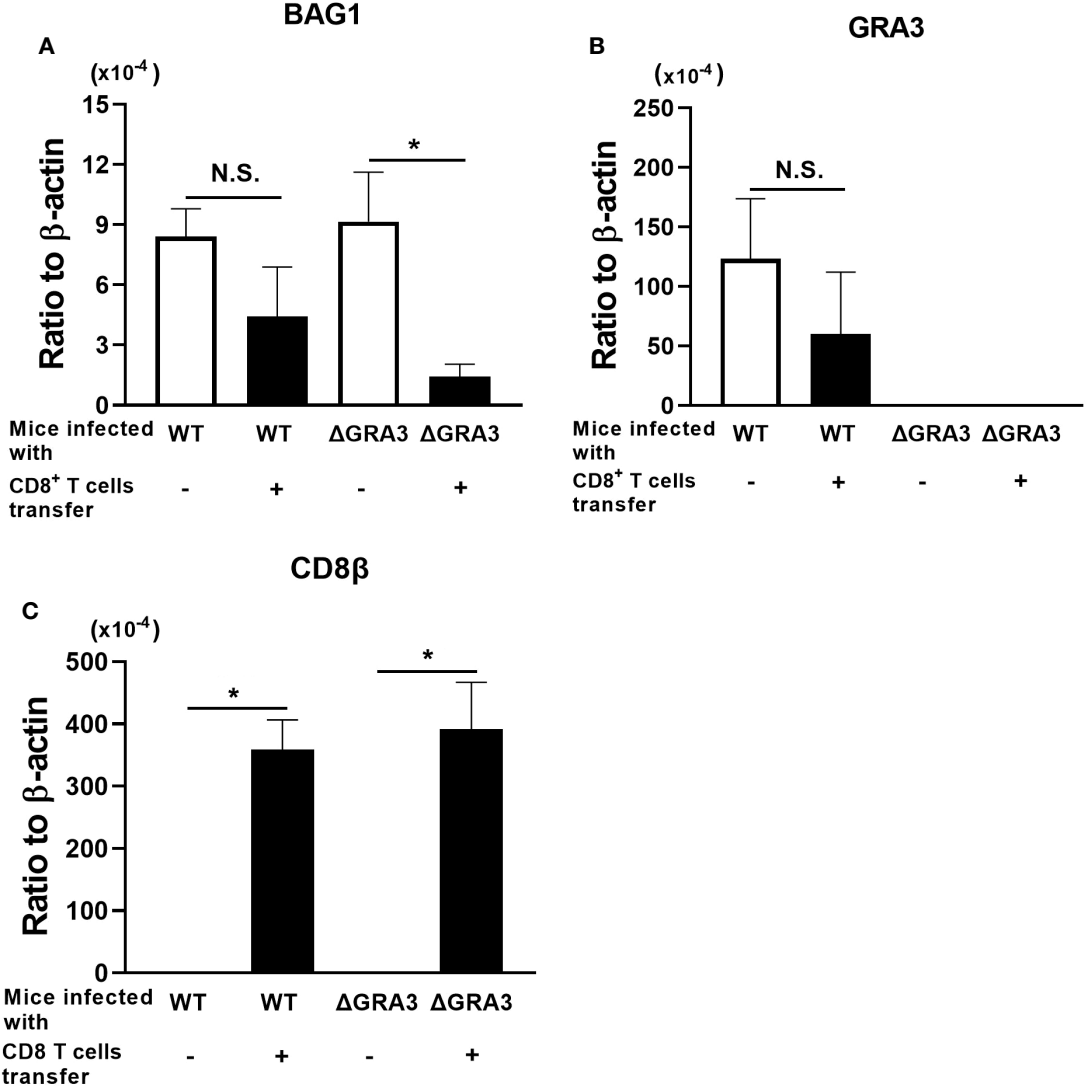
A deficiency in GRA3 in *T. gondii* facilitates an elimination of tissue cysts of the parasite by CD8^+^ immune T cells. SCID mice were infected intraperitoneally with 2 × 10^3^ tachyzoites of the WT Pru or ΔGRA3 strains of *T. gondii* and treated with sulfadiazine in drinking water (400 mg/L) beginning at 7 days after infection to establish chronic infection in their brains. At 3 weeks after the infection, mice received CD8^+^ immune T cells (3 × 10^6^ cells) purified from WT mice infected with the ME49 strain of *T. gondii*, intravenously from a tail vein. The Pru and ME49 strains both belong to the genotype II of the parasite. As a control, one group of each of the SCID mice infected with the WT Pru or ΔGRA3 strains did not receive any T cells. The mRNA levels for **(A)** BAG1 were measured in the brains of the recipients of the CD8^+^ immune T cells at 7 days after the T-cell transfer. These mRNA levels in the brains of the control SCID mice without the T-cell transfer were measured at 1 day before the time of the T-cell transfer to demonstrate the base levels of mRNA for these molecules at the time of the T-cell transfer and to determine how much of these mRNA levels decrease in the T-cell recipients at 7 days after the T-cell transfer. The mRNA levels for **(B)** GRA3 and **(C)** CD8β were also measured in the brains of these mice. Data represent the mean ± SEM. *p < 0.05.* N*.S.: Not significant.

The brains of the control group with no T-cell transfer in both the WT Pru- and ΔGRA3-infected SCID mice were obtained at 1 day before the T-cell transfer to indicate the cyst burden at the time of the T-cell transfer. The brains of the mice that received CD8^+^ immune T cells were obtained at 1 week after the T-cell transfer to determine the degrees of reduction of cerebral cyst burden when compared to the time of the T-cell transfer in the control group. Equivalent BAG1 mRNA levels were detected in the brains of the control group without the T-cell transfer in the WT Pru- and ΔGRA3-infected mice (**Figure 4A**). One week after the CD8^+^ immune T-cell transfer, the cerebral BAG1 mRNA levels in the ΔGRA3-infected mice were five times less than those in the control mice at the time of the T-cell transfer (*p* < 0.05, **Figure 4A**). In contrast, the cerebral BAG1 mRNA levels of the WT Pru-infected mice at 7 days after the CD8^+^ T-cell transfer were slightly greater than half of those of the control mice at the time of the T-cell transfer, and the difference did not reach statistical significance (*p* = 0.208, **Figure 4A**). These results indicate that ΔGRA3 *T. gondii* cysts have a significantly increased susceptibility to anti-cyst activity of CD8^+^ immune T cells for their elimination in the brain of infected mice.

To confirm the deficiency of GRA3 expression in ΔGRA3 *T. gondii* strain, we measured mRNA levels for GRA3 in the brains of SCID mice infected with the WT Pru and ΔGRA3 strains of the parasite. Large amounts of GRA3 mRNA were detected in the brains of the WT Pru-infected mice in parallel with those of BAG1 mRNA (**Figure 4B**). In the brains of the ΔGRA3-infected mice, GRA3 mRNA was somehow not completely absent but at the levels close to a detection limit (more than 3,500 times less than the levels detected in the WT Pru-infected mice) in the RT-PCR (**Figure 4B**). Even though trace amounts of GRA3 mRNA were detected in ΔGRA3 cysts, the marked reduction of the cyst burden in the ΔGRA3-infected SCID mice after receiving CD8^+^ immune T cells indicates that GRA3 plays an important role in the capability of *T. gondii* cysts to persist in the presence of anti-cyst CD8^+^ immune T cells.

In contrast to the notable differences in the reduction of BAG1 mRNA levels between the WT Pru- and ΔGRA3-infected SCID mice after receiving CD8^+^ immune T cells, the mRNA levels for CD8β did not differ between these two groups of mice (**Figure 4C**), suggesting that the transferred CD8^+^ immune T cells efficiently migrated into the brains of both WT Pru- and ΔGRA3-infected recipient mice in a similar manner. These results further support that GRA3 plays a critical role in the capability of *T. gondii* cysts to evade or resist anti-cyst CD8^+^ T-cell immunity and persist in the brains of infected hosts in the presence of those T cells.

The reason for the inefficient removal of the cysts of the WT Pru strain by CD8^+^ immune T cells in this study is unclear. One possible reason is that the CD8^+^ immune T cells were purified from mice infected with the ME49 strain, whereas the recipient SCID mice were infected with the Pru strain. Although both the Pru and ME49 strains belong to the genotype II of *T. gondii* as mentioned earlier, there could be some variations in the amino acid sequences and structures of a part of the parasite molecules that anti-cyst CD8^+^ T cells recognize, between these two strains of the parasite. These differences may have reduced the efficiencies of the CD8^+^ immune T cells obtained from mice infected with the ME49 strain to eliminate the cysts of the Pru strain.

## Discussion

The tissue cysts are the basis of the persistence of *T. gondii* during the chronic stage of infection. The cyst stage of the parasite is able to persist in immunocompetent hosts during the chronic stage of infection. Although CD8^+^ T cells of infected mice have the capability to recognize the cyst-harboring cells and eliminate a majority of them through perforin-mediated mechanisms (3, 5, 23), small potions of the cysts are able to avoid their elimination by the T cells and persist in the presence of the anti-cyst T-cell immunity (3, 5, 23) through unknown mechanisms. The present study uncovered that the selected population of *T. gondii* cysts that successfully persisted in the brains of infected SCID or RAG1^−/−^ mice after an adoptive transfer of perforin-sufficient CD8^+^ immune T cells have markedly increased mRNA expression levels of five secretory proteins, GRA1, GRA2, GRA3, GRA7, and ROP35, among eight GRAs (GRA1–GRA8) and five ROPs (ROP5, ROP16–ROP18, and ROP35) tested. Notably, such increased expressions of these selected secretory proteins did not occur following a transfer of normal CD8^+^ T cells from uninfected mice or CD8^+^ immune T cells from infected Prf1^−/−^ mice. These observations provided the novel evidence that *T. gondii* cysts that successfully persisted in the presence of the perforin-mediated anti-cyst CD8^+^ T-cell immunity are a uniquely selected population that have notably increased mRNA expressions for those selected GRA and ROP proteins when compared to the general population of cysts that exist in the absence of the protective immunity.

In contrast to the selected secretory molecules, GRA1, GRA2, GRA3, GRA7, and ROP35, whose mRNA levels are notably upregulated in the cysts that persisted in the presence of anti-cyst CD8^+^ immune T cells, the present study also revealed that mRNA levels for the non-secretory control molecule, SAG2C, expressed on the surface of bradyzoites were not elevated in those cysts capable of persisting under the host immunity. A recent study demonstrated that a mutant strain of *T. gondii* with a deletion of the *SAG2CDYX* cluster of genes maintain significantly fewer cysts in the brains of infected mice at 4 and 9 weeks after infection (22). Since a previous study showed that *T. gondii*-infected humans do not induce IgG antibody responses to SAG2C (25), the presence of the fewer cysts in the SAG2CDYX-deletion mutant strain in infected mice may not be related to the immune evasion of *T. gondii cysts* for their persistence during the chronic stage of infection.

Among the five selected secretory proteins, GRA1, GRA2, GRA3, GRA7, and ROP35, the present study identified that the degree of increased mRNA expression levels for only GRA1, GRA3, and ROP35 in the cysts significantly correlated with their successful persistence of *T. gondii* cysts through the condition in which greater portions of cysts from their total population are eliminated by CD8^+^ immune T cells. Notably, our study further revealed that the tissue cysts of the ΔGRA3 mutant strain of *T. gondii* have an increased susceptibility to the anti-cyst activity of CD8^+^ immune T cells and display their accelerated elimination from the brains of infected mice by T cells. To our knowledge, *T. gondii* molecules that mediate the capability of tissue cysts to persist in the presence of the protective immunity have not been reported before.

GRA3 is present within both the PV and the cyst wall (9, 18). A recent study (16) demonstrated that a deficiency in GRA3 results in a marked reduction of cyst numbers in the brains of infected mice, and suggested that GRA3 plays a structural or organizational role during cyst development or in cyst maintenance. Therefore, it is possible that increased levels of GRA3 enhance the integrity of the cyst wall and contribute to resisting the anti-cyst activity of CD8^+^ T cells. Another recent study (26) using *in vitro* cultures of bone-marrow-derived macrophages and dendritic cells infected with ovalbumin (OVA)-expressing tachyzoites demonstrated that these antigen-presenting cells infected with OVA-expressing tachyzoites deficient in GRA3 stimulate OVA-specific CD8^+^ T cells more potently than the antigen-presenting cells infected with the OVA-expressing wild-type control tachyzoites, indicating an immunosuppressive activity of GRA3 to downregulate the antigen-presenting activity of infected macrophages and dendritic cells to activate CD8^+^ T cells. Thus, GRA3 present in *T. gondii* cysts may also play a similar pathogenic role in cyst-harboring cells to suppress their antigen presentation activity for avoiding their recognition by anti-cyst CD8^+^ T cells. Similarly, another recent study (27) using tachyzoites demonstrated that GRA3 located in the PV interacts with the Golgi, leading to the formation of tubules and the entry of host Golgi materials into the PV and dysregulation of anterograde transport in infected cells. The Golgi network plays critical roles in antigen presentation by the major histocompatibility complex (MHC) class I molecules to stimulate CD8^+^ T cells. Therefore, it is possible that GRA3 located in the cyst wall interacts with the Golgi in a similar manner as this molecule in the PV does and inhibits the antigen presentation by the MHC class I molecules to evade the anti-cyst activity of CD8^+^ T cells. It is also possible that the GRA3-mediated disruption of the Golgi transport suppresses the secretion of immune molecules critical for inducing anti-cyst activity of CD8^+^ T cells.

The results in the present study do not exclude a possible involvement of *T. gondii* molecules other than GRA3 in the immune evasion of the cysts to avoid the attack by CD8^+^ immune T cells. GRA1, GRA2, GRA7, and ROP35 could be those candidate molecules that have a potential to be involved in the immune evasion of *T. gondii* cysts. There are currently no drugs available to target the cyst stage of *T. gondii*. One-third of the human population worldwide is estimated be chronically infected with this parasite (1) as mentioned earlier. It is well appreciated that the tissue cysts are the source of reactivation of the infection with this parasite, which causes the development of life-threatening toxoplasmic encephalitis in immunocompromised patients (1). In addition, even in immunocompetent individuals, recent epidemiological studies reported increased incidence of cancers in the individuals who are seropositive to (infected with) *T. gondii* when compared to seronegative (uninfected) people (28, 29). Therefore, it is critical to develop a method that allows us to target *T. gondii* cysts for their elimination and a cure for the chronic infection with this parasite. The evidence in the present study has the potential to provide a valuable basis to begin understanding how *T. gondii* cysts are able to persist in the presence of the anti-cyst protective T-cell immunity during the chronic stage of infection and to design a novel method for disrupting the immune evasion mechanism(s) of *T. gondii* cysts and their eradication to eliminate this widespread chronic infection.

## Data availability statement

The original contributions presented in the study are included in the article/supplementary material. Further inquiries can be directed to the corresponding author.

## Ethics statement

The animal study was approved by Institutional Animal Care and Use Committee of University of Kentucky. The study was conducted in accordance with the local legislation and institutional requirements.

## Author contributions

YS: Conceptualization, Data curation, Formal Analysis, Funding acquisition, Investigation, Project administration, Supervision, Validation, Visualization, Writing – original draft, Writing – review & editing. RM: Formal Analysis, Investigation, Visualization, Writing – review & editing. MA: Investigation, Writing – review & editing. EO: Investigation, Writing – review & editing. QS: Investigation, Writing – review & editing. BF: Investigation, Writing – review & editing. DB: Supervision, Writing – review & editing.
